# Emotional information affects fission illusion induced by audio-visual interactions

**DOI:** 10.1038/s41598-020-57719-y

**Published:** 2020-01-22

**Authors:** Yasuhiro Takeshima

**Affiliations:** Department of Psychology, Doshisha University, Doshisha, Japan

**Keywords:** Sensory processing, Human behaviour

## Abstract

Multisensory integration is affected by various types of information coming from different sensory stimuli. It has been suggested that emotional information also influences the multisensory integration process. The perceptual phenomena induced by audio-visual integration are modulated by emotional signals through changing individuals’ emotional states. However, the direct effects of emotional information, without changing emotional states on the multisensory integration process have not yet been examined. The present study investigated the effects of an emotional signal on audio-visual integration. The experiments compared the magnitude of audio-visual fission and fusion illusions using facial expression stimuli and simple geometric shapes. Facial expression stimuli altered the criterion difference for discerning the number of flashes when two beeps were simultaneously presented in Experiment 1. These stimuli did not affect the fission illusion’s magnitude. For simple geometric shapes, emotional shapes perceptually induced a larger fission illusion in Experiment 2. The present study found that the emotional valence included in simple geometric shapes induced a larger fission illusion. Moreover, current results suggest that emotional faces modulate response criterion for fission illusion in discernment of the number of flashes. Future studies should elucidate in detail the mechanism of emotional valence effects on audio-visual integration.

## Introduction

Multisensory integration is an important function in the perception of an external environment. Many studies have examined the multisensory information integration process reporting that multisensory percepts are stable and salient compared to uni-sensory perception^1^. In particular, the process of integrating visual and auditory information (i.e., audio-visual integration) has been reported in the facilitatory aspects of this interaction. For example, auditory stimuli enhance the perceived intensity of visual stimuli^2^. Moreover, the detection sensitivity of the visual target becomes higher when presentations of visual and auditory stimuli are spatially and/or temporally consistent^3,4^.

Emotional information affects the audio-visual integration process. For example, Maiworm *et al*.^5^ have shown that the ventriloquism effect^6^ was reduced using a preceding task of sound source localization using fearful voices. Moreover, Kitamura *et al*.^7^ have reported that task-irrelevant happy background music extended the temporal binding window for audio-visual stimuli in a stream/bounce display^8^ for participants with lower depressive tendencies. However, in these studies, emotional signals altered the perceptual phenomena induced by audio-visual integration, by changing individuals’ emotional states. In other words, these studies examined the effects of emotional information on audio-visual interactions by using task-irrelevant emotional stimuli. Negative^9,10^ and positive^11,12^ emotional stimuli rapidly and strongly attract attention. Audio-visual integration could be modulated by visual attention^13,14^. Therefore, emotional information from visual stimuli could directly affect the audio-visual integration process.

Therefore, the present study examined the effects of emotional information by using task-relevant emotional stimuli, without changing individual’s emotional states, during the audio-visual integration process. In the experiments, task-relevant emotional stimuli were presented to compare the illusory phenomenon induced by audio-visual interaction. Fission and fusion illusions have been used to measure susceptibility to multisensory integration^15,16^. When a brief single flash is accompanied by two simultaneous beeps, two flashes are often perceived: this phenomenon is called the fission illusion^17,18^. In contrast, when two brief flashes are accompanied by one simultaneous beep, a single flash is often perceived: this is called the fusion illusion^19^. Several fMRI studies have reported that the activation of primary visual cortex (V1) during the fission illusion is similar to the response elicited by the presentation of two physical flashes and the response during the fusion illusion is similar to that elicited by the presentation of one physical flash^20,21^.

Various characteristics of visual stimuli modulate the audio-visual integration process as demonstrated by the fission illusion. For example, visual complexity and spatial frequency modulate the occurrence rates of a fission illusion^22,23^. The processing speed for visual stimuli, which is controlled by visual complexity or spatial frequency, would affect fission illusion processing^22,23^. Moreover, it is difficult to induce a fission illusion with images of familiar faces and buildings^14^. While familiarity is a higher-level characteristic of visual stimuli, it influences the early stages of audio-visual integration^24^. However, these visual characteristics only affected the fission illusion, not the fusion illusion. Fission and fusion illusions have different underlying mechanisms^25^. Compared with the fission illusion, the fusion illusion is strongly reflective of individual differences in visual processing^26^. Therefore, it is possible that emotional information could affect the fission illusion process.

In the present study, two types of emotional stimuli were used as visual stimuli: facial expressions and simple geometric shapes. Facial expression stimuli are typical emotional stimuli and have been used in various behavioural tasks^27,28^. Additionally, simple geometric shapes have been reported to convey emotional valence^29–33^, and have also been used in behavioural tasks^34,35^. By using these types of emotional stimuli, the present study attempted to widely examine the effects of emotional information with task-relevant stimuli on the audio-visual integration process that occur during fission and fusion illusions.

## Results

### **Facial expression stimuli (Experiment 1)**

**Figure 1:**
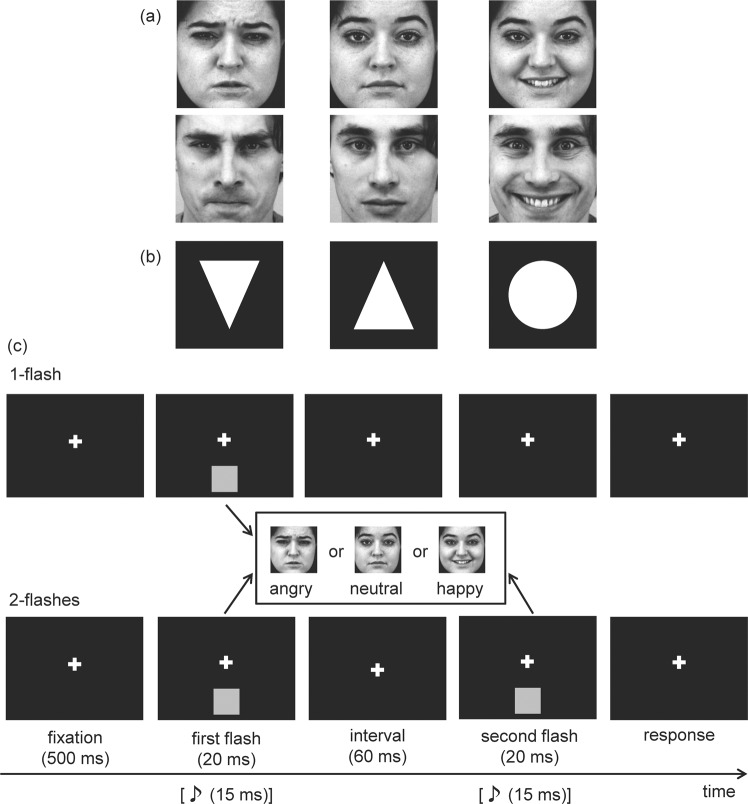
The visual stimuli and schematic representation of the procedure used in the present experiments. (**a**) Visual stimuli used in Experiment 1. Faces were selected from the Karolinska Directed Emotional Faces Database^58^. (**b**) Visual stimuli used in Experiment 2. Angry face and downward-pointing triangle were rated as negative stimuli, neutral face and upward-pointing triangle were rated as neutral stimuli, and happy face and circle were rated as positive stimuli. (**c**) The top panels indicate the 1-flash sequence. The bottom panels indicate the 2-flashes sequence. The grey square indicates the position of presented visual stimulus.

Experimental settings were based on Takeshima and Gyoba^22,23^. Three types of facial expression stimuli (neutral, angry, and happy faces) were presented as visual stimulus. Visual stimulus was presented below the fixation point. In the trial, a white fixation cross (0.5 × 0.5° visual angle) was presented for 500 ms followed by the presentation of visual stimuli once or twice for 20 ms each (Figure 1). The auditory stimulus was a pure tone (3500 Hz and 80 dB(A)). The duration of the auditory stimulus was 15 ms (including ramp times of 2.5 ms at the start and end of the sound wave envelope). Auditory stimulus was presented once or twice, and without sound as a baseline. The stimulus onset asynchrony (SOA) between the first and second stimulus presentations was 80 ms. The participants’ task was to discern the number of flashes. After this main task, participants rated the facial stimuli by using a seven-point bipolar semantic differential scale^36^ that included four items^37–39^.

First, emotional valence scores were calculated for each facial expression by averaging the actor’s gender and four semantic differential dimensions (Table 1). A one-way analysis of variance (ANOVA) with face (3) as the within-participants factor was conducted. The results revealed a significant main effect (*F* (2, 40) = 30.20, *p* <  = 0.001, η_p_^2^ = 0.60). Multiple comparisons (Shaffer’s modified sequentially rejective Bonferroni procedure by Donoghue’s S2 algorithm^40,41^) indicated that the angry face received lower scores than the neutral (*t* (20) = 5.49, *p* <  = 0.001, *d* = 1.51) and happy faces (*t* (20) = 6.43, *p* <  = 0.001, *d* = 2.53). The rating score for the happy face was higher than that for the neutral face (*t* (20) = 3.72, *p* = 0.001, *d* = 1.14). The neutral, angry, and happy faces were thus rated as neutral, negative, and positive stimuli, respectively.

**Table 1:** Emotional valence scores for visual stimuli in Experiment 1. Values in parentheses indicate standard errors of the mean (*N* = 21).

adjective pairs		faces		
low	high	neutral	angry	happy
unfriendly	friendly	3.69 (0.25)	2.60 (0.22)	4.88 (0.31)
cruel	kind	3.74 (0.27)	3.56 (0.21)	5.12 (0.23)
unpleasant	pleasant	3.98 (0.18)	2.62 (0.22)	4.62 (0.23)
bad	good	4.07 (0.17)	2.79 (0.19)	4.90 (0.26)
valence rating (Mean)		3.92 (0.16)	2.89 (0.14)	4.88 (0.20)

For the discernment of number of flash, error rates were calculated for each condition (Table 2). Moreover, the d-prime (*d’*) and criterion (*c*) scores used to discriminate the number of flashes were calculated for each condition according to signal detection theory^42^. The *d’* and *c* scores were calculated separately when no-beep, 1-beep, or 2-beeps were presented under each face condition same as previous studies^25,43^. The *d’* and *c* scores are shown in Table 3. Furthermore, *d’*-illusion and *c*-difference scores^44,45^ were calculated by subtracting *d’* and *c* scores with 1-beep/2-beeps from those with no-beep, respectively. The difference between 2-beeps and no-beep reflect the index associated with fission illusion, whereas that between 1-beep and no-beep reflects the index associated with fusion illusion.

**Table 2:** Error rates of each face in Experiment 1. The values of mean fission and fusion illusion rates in 1-flash|2-beeps and 2-flashes|1-beep conditions, respectively. Values in parentheses indicate standard errors of the mean (*N* = 21).

faces	no-beep		1-beep		2-beeps	
	1-flash	2-flashes	1-flash	2-flashes	1-flash	2-flashes
neutral	0.13 (0.03)	0.07 (0.02)	0.03 (0.01)	0.45 (0.08)	0.68 (0.07)	0.03 (0.01)
angry	0.15 (0.03)	0.10 (0.03)	0.05 (0.02)	0.39 (0.08)	0.73 (0.06)	0.04 (0.01)
happy	0.12 (0.03)	0.12 (0.03)	0.03 (0.01)	0.43 (0.07)	0.71 (0.07)	0.03 (0.02)

**Table 3:** D-prime and criterion scores under each face in Experiment 1. Values in parentheses indicate standard errors of the mean (*N* = 21).

faces	no-beep		1-beep (fusion)		2-beeps (fission)	
	*d’*	*c*	*d’*	*c*	*d’*	*c*
neutral	3.13 (0.21)	−0.18 (0.12)	2.19 (0.26)	0.91 (0.13)	1.32 (0.27)	−1.01 (0.05)
angry	2.90 (0.19)	−0.14 (0.13)	2.17 (0.31)	0.76 (0.16)	1.13 (0.25)	−1.42 (0.13)
happy	2.93 (0.18)	0.01 (0.14)	2.22 (0.22)	0.89 (0.14)	1.22 (0.22)	−1.44 (0.16)

**Figure 2:**
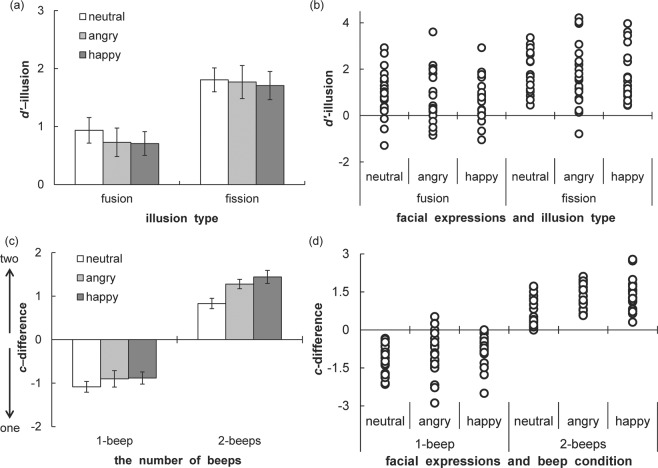
The d’-illusion and *c*-difference scores of Experiment 1. The *d’*-illusion and *c*-difference scores were calculated by subtracting *d’* and *c* scores with 1-beep/2-beeps from those with no-beep, respectively. (**a**) Bar graph with mean *d’*-illusion scores and error bar with SEM (*N* = 21). (**b**) Scatterplots of individuals’ data of *d’*-illusion scores. (**c**) Bar graph with mean *c*-difference scores and error bar with SEM (*N* = 21). (**d**) Scatterplots of individuals’ data of *c*-difference scores.

The *d’*-illusion and *c*-difference scores of Experiment 1 are shown in Figure 2. A one-way ANOVA with face (3) as the within-participant factor was separately conducted for *d’*-illusion in 2-beeps (i.e., fission illusion) and 1-beep (i.e., fusion illusion) conditions, because the comparison between fission and fusion illusion magnitudes was not included in the purpose of this study. The main effects of face were not significant in fission (*F* (2, 40) = 0.76, *p* = 0.48, η_p_^2^ = 0.04) and fusion (*F* (2, 40) = 0.12, *p* = 0.89, η_p_^2^ = 0.01) illusions. *D’*-illusion scores were positive for all three facial stimuli in fission and fusion conditions. Thus, fission and fusion illusions indeed occurred for this experiment. The magnitudes did not differ among the facial expressions in both fission and fusion illusion. The magnitude of the fission illusion did not differ among the facial expressions.

A one-way ANOVA with face (3) as the within-participant factor was separately conducted for *c*-difference in 1-beep and 2-beeps conditions. In the fission illusion, the main effect of face was significant (*F* (2, 40) = 11.45, *p* <= 0.001, η_p_^2^ = 0.36). Multiple comparisons indicated that *c*-difference was higher in in angry (*t* (20) = 3.29, *p* = 0.004, *d* = 0.86) and happy (*t* (20) = 4.24, *p* = 0.001, *d* = 0.99) faces than in neutral faces. Thus, emotional faces (i.e., both angry and happy) had a larger tendency to respond to 2-flashes in fission illusion compared to a neutral face. This result suggests that the emotional information in facial expressions affected the audio-visual integration process at the higher-order level when the number of beeps was two. In contrast, the main effect was not significant in the fusion illusion (*F* (2, 40) = 1.72, *p* = 0.192, η_p_^2^ = 0.08), indicating that emotional faces did not affect the response bias when there was 1-beep.

Finally, the correlation between the amounts of changing valence and *d’*-illusion (calculated by subtracting the angry and happy faces from the neutral face on valence and *d’*-illusion) was complementarily computed. The correlation scores were not significant in both fission (*r* (40) = 0.05, *p* = 0.714) and fusion (*r* (40) = −0.08, *p* = 0.639) illusions.

### **Simple geometric shapes (Experiment 2)**

Three types of simple geometric shapes (upward-pointing triangle, downward-pointing triangle, and circle) were presented instead of facial expression stimuli in Experiment 2. As in Experiment 1, emotional valence scores were calculated for each simple geometric shape by averaging the four semantic differential dimensions (Table 4). A one-way ANOVA with shape (3) as the within-participants factor was conducted. The results revealed a significant main effect (*F* (2, 42) = 47.68, *p* <= 0.001, η_p_^2^ = 0.69). Multiple comparisons indicated that the downward-pointing triangle received lower scores than both the upward-pointing triangle (*t* (21) = 4.31, *p* <  = 0.001, *d* = 1.37) and circle (*t* (21) = 6.75, *p* <  = 0.001, *d* = 1.56). The rating score for the circle was higher than the score for the upward-pointing triangle (*t* (21) = 8.54, *p* <  = 0.001, *d* = 1.92). Thus, the upward-pointing triangle, downward-pointing triangle, and circle were rated as neutral, negative, and positive stimuli, respectively. This rating tendency for simple geometric shapes was almost the same as in the previous studies^29,34^.

**Table 4:** Emotional valence scores for visual stimuli in Experiment 2. Values in parentheses indicate standard errors of the mean (*N* = 22).

adjective pairs		shapes		
low	high	upward-pointing triangle	downward-pointing triangle	circle
unfriendly	friendly	4.72 (0.23)	2.91 (0.23)	5.73 (0.30)
cruel	kind	3.77 (0.22)	2.82 (0.22)	5.91 (0.25)
unpleasant	pleasant	4.41 (0.22)	3.59 (0.22)	5.73 (0.22)
bad	good	4.68 (0.27)	3.50 (0.27)	5.82 (0.24)
valence rating (Mean)		4.40 (0.18)	3.20 (0.20)	5.80 (0.20)

**Figure 3:**
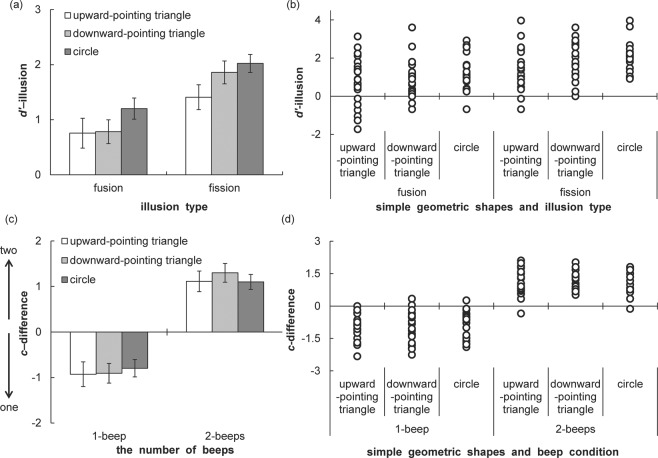
The *d*’-illusion and *c*-difference scores of Experiment 2. The *d’*-illusion and *c*-difference scores were calculated by subtracting *d’* and *c* scores with 1-beep/2-beeps from those with no-beep, respectively. (a) Bar graph with mean *d’*-illusion scores and error bar with SEM (*N* = 22). (b) Scatterplots of individuals’ data of *d’*-illusion scores. (c) Bar graph with mean *c*-difference scores and error bar with SEM (*N* = 22). (d) Scatterplots of individuals’ data of *c*-difference scores.

For the discernment of number of flash, error rates were calculated for each condition (Table 5). Moreover, the *d’* and *c* scores were calculated for each condition. The *d’* and *c* scores are shown in Table 6. Moreover, *d’*-illusion and *c*-difference scores were computed by subtracting *d’* and *c* scores with 1-beep or 2-beeps from those with no-beep, respectively (Figure 3). A one-way ANOVA with shape (3) as the within-participant factor was separately conducted for *d’*-illusion in fission and fusion illusions. In the fission illusion, the main effect of shape was significant (*F* (2, 41) = 5.23, *p* = 0.009, η_p_^2^ = 0.20). Multiple comparisons indicated that *d’*-illusion was higher in the downward-pointing triangle (*t* (21) = 2.22, *p* = 0.038, *d* = 0.44) and circle (*t* (21) = 3.25, *p* = 0.004, *d* = 0.66) than in the upward-pointing triangle. On the other hand, the main effect of shape was not significant in the fusion illusion (*F* (2, 41) = 2.22, *p* = 0.012, η_p_^2^ = 0.10). *D’*-illusion scores were positive for all three simple geometric shapes in both fission and fusion conditions. Thus, fission and fusion illusions occurred in this experiment. Moreover, the magnitude of the fission illusion was higher in both negative and positive emotional shapes. However, illusory magnitude did not differ among simple geometric shapes in the fusion illusion.

**Table 5:** Error rates of each shape in Experiment 2. The values of mean fission and fusion illusion rates in 1-flash|2-beeps and 2-flashes|1-beep conditions, respectively. Values in parentheses indicate standard errors of the mean (*N* = 22).

	no-beep		1-beep		2-beeps	
shapes	1-flash	2-flashes	1-flash	2-flashes	1-flash	2-flashes
upward-pointing triangle	0.11 (0.03)	0.07 (0.02)	0.02 (0.01)	0.39 (0.07)	0.58 (0.07)	0.02 (0.01)
downward-pointing triangle	0.12 (0.03)	0.06 (0.02)	0.03 (0.01)	0.34 (0.07)	0.68 (0.06)	0.01 (0.01)
circle	0.08 (0.03)	0.07 (0.02)	0.03 (0.01)	0.41 (0.06)	0.65 (0.07)	0.02 (0.01)

**Table 6:** D-prime and criterion scores under each face in Experiment 2. Values in parentheses indicate standard errors of the mean (*N* = 22).

	no-beep		1-beep (fusion)		2-beeps (fission)	
shapes	*d’*	*c*	*d’*	*c*	*d’*	*c*
upward-pointing triangle	3.24 (0.24)	−0.10 (0.09)	2.49 (0.22)	0.83 (0.13)	1.83 (0.26)	−1.21 (0.16)
downward-pointing triangle	3.33 (0.21)	−0.18 (0.10)	2.55 (0.23)	0.72 (0.15)	1.48 (0.23)	−1.48 (0.14)
circle	3.51 (0.16)	0.05 (0.12)	2.31 (0.23)	0.84 (0.11)	2.31 (0.23)	−1.05 (0.05)

A one-way ANOVA with shape (3) as the within-participant factor was separately conducted for *c*-difference under 1-beep and 2-beeps conditions. The main effects of shape were not significant in both fission (*F* (2, 41) = 1.23, *p* = 0.30, η_p_^2^ = 0.06) and fusion (*F* (2, 41) = 0.54, *p* = 0.58, η_p_^2^ = 0.03) illusions. Thus, the magnitude of response biases did not differ among simple geometric shapes in both fission and fusion illusions. The current experiment showed that the emotional valence of simple geometric shapes modulates audio-visual integration at the perceptual level.

Finally, the correlation between the amounts of changing valence and *d’*-illusion (calculated by subtracting the downward-pointing triangle and circle from the upward-pointing triangle on valence and *d’*-illusion) was complementarily computed. The correlation scores were not significant in both fission (*r* (42) = 0.21, *p* = 0.169) and fusion (*r* (42) = 0.13, *p* = 0.411) illusions.

## Discussion

The present study examined the effects of emotional information on audio-visual integration. Facial expression stimuli and simple geometric shapes with emotional valence were presented as visual stimuli, and the magnitudes of both fission and fusion illusions were compared across these stimuli. Fission and fusion illusions were observed in both experiments. For the facial expression stimuli in Experiment 1, angry and happy faces largely shifted to a two flashes response compared to neutral face when the number of auditory beeps was two. Therefore, emotional faces were strongly affected by the number of beeps in the discernment of the number of flashes. For the simple geometric shapes, the *d’*-illusion scores of the downward-pointing triangle and circle were larger than that for the upward-pointing triangle for the fission illusion in Experiment 2. Thus, emotional information within simple geometric shapes facilitated the occurrence of the fission illusion at the perceptual level. On the other hand, emotional stimuli did not affect the *d’*-illusion for the fusion illusion and the *c*-difference for 1-beep under both facial expression stimuli and simple geometric shapes.

When two beeps were simultaneously presented with one visual stimulus, emotional faces modulated the response criterion for fission illusion. Thus, the facial expression stimuli modulated the processing of audio-visual integration at the higher-order level. In simple geometric shapes, particularly in the downward-pointing triangle, emotional valence is perceived through visual features: V-shapes^34^. On the other hand, the perceptual mechanisms of emotional valence would be more complex in facial expression stimuli than in simple geometric shapes. Thus, facial expression stimuli would affect audio-visual integration processing of complex visual features at the higher-order level compared to simple geometric shapes.

In the simple geometric shapes, emotional information induced larger fission illusions at the perceptual level. The modulation of the fission illusion is likely associated with attention. Selective attention enhances the neural processes associated with the fission illusion^46^. Moreover, attention to one sensory modality can spread to another sensory modality and enhance multisensory integration processing^13,14^. Negative^9,10^ and positive^11,12^ stimuli strongly attract attention. In particular, the saliency of emotional stimuli^47,48^ might attract attention. Many phenomena have been reported with respect to attracting and modulating attention by emotional information^49^. Thus, the attention attracted due to emotional valence might modulate the magnitude of the fission illusion in the present study. The other factors besides emotional valence are also related to the larger fission illusion of emotional shapes. The illusion’s magnitudes were almost the same between the negative and positive stimuli. However, attentional bias is higher for negative than for positive stimuli^50^. Thus, the current effects of positive stimuli might include extending the temporal binding window for audio-visual stimuli via positive emotion^7^.

It is necessary to controversially discuss the interpretations associated with attention. Two types of attention are related to the multisensory integration process: modality-specific and cross-modal attention^51,52^. Talsma *et al*.^52^ have proposed that bottom-up (i.e., stimulus-driven) mechanisms induced by the interaction between sensory modalities automatically bring attention towards multisensory events. In the current experiments, the experimental task was to discern the number of flashes. Therefore, participants’ attention would be mainly directed towards visual stimulus, and then spread to multisensory events. For simple geometric shapes with emotional valence, strongly stimulus-driven attention might have induced the larger fission illusion by directing attention towards multisensory events^46^. However, this study could not directly test how emotional information modulated attention. This limitation should be an endeavor pursued in future studies.

One of the limitations of this study is that the different results between the fission and fusion illusions could not be elucidated. Whereas the fusion illusion also occurred in the experiments, emotional information conveyed with facial expressions and simple geometric shapes did not affect the magnitude of the fusion illusion and the criterion difference. Previous studies suggest that the mechanisms of the fusion illusion differ from those of the fission illusion^25,26^. An ERP study reported that the fusion illusion is associated more with post-perceptual processing^53^. Therefore, there are many different mechanisms between fission and fusion illusions. These differences in the underlying mechanisms could be attributed to the different results of the current study between fission and fusion illusions. However, the current study could not elucidate the critical factors underlying the different results between fission and fusion illusions. Further researches are necessary to analyze this limitation.

Another limitation was the facilitation of the magnitude of the fission illusion included in the effects of emotional information and other factors together with the simple geometric shapes. The correlation between the amounts of changing valence and *d’*-illusion was computed, and revealed non-significant correlation scores in Experiment 2. Thus, the facilitation of simple geometric shapes in creating the fission illusion could not be explained by emotional valence alone. Several visual features (e.g., peripheral/central luminance) differed among simple geometric shapes in current study. These visual features should be more strictly controlled. In the future, it is necessary to clarify the effects of other factors along with simple geometric shapes.

The present study found that task-relevant emotional stimuli affect audio-visual integration by using fission and fusion illusions. In audio-visual integration, facial expression stimuli modulate the response criterion for audio-visual illusion whereas simple geometric shapes with emotional valence facilitate the magnitude of the audio-visual illusion. The present findings support the relationship between multisensory integration and emotions. However, this study was unable to elucidate several problems. For example, similar experiments should be conducted on the fission illusion caused by visual^54^ and audio-visual inducers^55^. Moreover, the neural mechanism underlying the present effects has not been clarified. For all of these reasons, future studies should build on the present work.

## Method

### Ethical statement

These experiments were approved by the ethics committee of Doshisha University (No. 17013) and were performed in accordance with the approved guidelines and the Declaration of Helsinki. All participants gave written informed consent before participating.

### **Participants**

Twenty-one (10 women and 11 men; mean age = 20.86 ± 1.06 years) and 22 (8 women and 14 men; mean age = 23.05 ± 2.98 years) observers participated in Experiment 1 and Experiment 2, respectively. All of the participants orally reported normal or corrected-to-normal vision and normal hearing. Participants were given 500 Japanese yen for their participation.

### **Apparatus**

Stimuli were generated and controlled by means of a custom-made program, written using MATLAB (The MathWorks, Inc.), Psychtoolbox^56–58^, and a laptop PC (MacBook Pro, Apple Inc.). The visual stimuli were displayed on a 21-inch CRT-display (Trinitron CPD-G520, Sony; resolution: 1024 × 768 pixels; refresh rate: 100 Hz). The auditory stimuli were conveyed through an audio interface (Clarett 2Pre, Focusrite) and headphones (MDR-CD900ST, Sony). The simultaneity of the visual and auditory stimuli was confirmed using a digital oscilloscope (DS-5424A, Iwatsu). The experiment was conducted in a slightly darkened room with 37.5 dB (A) of background noise. Participants viewed the monitor binocularly at a distance of 70 cm with their heads stabilized on a chin rest.

### **Stimuli**

In Experiment 1, the visual stimuli consisted of three facial expression categories (neutral, angry, and happy). Two actors’ images (image id: F03ANS, F03NES, F03HAS, M25ANS, M25NES, and M25HAS) were selected from the Karolinska Directed Emotional Faces Database^59^ according to both emotional intensity and valence ratings^60^. In this study, only these two images were used in consideration of the load of the participants’ rating valence. All facial images were converted to grayscale and cropped into a square (2.0 × 2.0° visual angle) to remove visual features outside of the face. In Experiment 2, three types of simple geometric shapes (upward-pointing triangle, downward-pointing triangle, and circle) were presented. Armbruster *et al*.^29^ have shown that a downward-pointing triangle is perceived as unpleasant while a circle is perceived as pleasant by using skin conductance response. The size of each visual stimulus was within a 2.0 × 2.0° rectangle and colour of these stimuli was white. All stimuli and the white fixation cross (0.5 × 0.5°) were presented on a black background. The duration of the visual stimuli was 20 ms. Visual stimulus was presented below the fixation point. The vertical distance between the fixation point and the centre of the visual stimulus was 6.0° (5.0° eccentricity). The visual stimulus was presented once (1-flash) or twice (2-flashes) during each trial. The auditory stimulus was a pure tone at a frequency of 3500 Hz. The duration of the auditory stimulus was 15 ms (including ramp times of 2.5 ms at the start and end of the sound wave envelope), and the sound pressure level of the stimulus was 80 dB (A). The experimental condition for the auditory stimulus comprised three levels: no-beep, 1-beep, or 2-beeps. No-beep indicates an absence of beep sounds, 1-beep means that one beep was presented during the first flash period, and 2-beeps denotes that beeps were presented twice (during both the first and second flash periods). The stimulus onset asynchrony (SOA) between the first and second stimulus presentations was 80 ms.

### **Procedure**

All trials were initiated by pressing the ‘0’ key on a keyboard at each participants’ own pace. Each trial consisted of a 500 ms fixation followed by the presentation of visual stimuli once or twice. Participants were instructed to report the number of flashes they perceived by pressing one of two keys: ‘1’ or ‘2’ for one or two flashes. The experiment followed a 3 (face / shape: angry, happy, or neutral / downward-pointing triangle, circle, or upward-pointing triangle) × 3 (beep: no-beep, 1-beep, or 2-beeps) design. Each participant completed 360 trials: 3 faces × 3 beeps × 2 the number of visual flashes once (1-flash) or twice (2-flashes) × 20 repetitions. For each participant, all response data was accurately collected, and thus data reduction was not conducted in the current experiments. Data from total of 7520 (Experiment 1: 21 participants × 360 trials) and 7920 (Experiment 2: 22 participants × 360 trials) trials were used for analysis.

After the above task, participants rated the visual stimuli used in the experiments. Each participant was given a booklet depicting one face or shape on each page. The sizes of these stimuli were 3.0 × 3.0 cm. Participants were asked to rate each face in terms of bad–good, unpleasant–pleasant, unfriendly–friendly, and cruel–kind^37–39^ using a seven-point bipolar semantic differential scale^41^. Lower numbers reflected more negative ratings.

### **Analysis**

For subjective rating scores, emotional valence scores were calculated for each facial expression/shape by averaging four semantic differential dimensions (Tables 1 and 4). In Experiment 1, the scores of the actor’s gender were also averaged. A one-way ANOVA with face/shape (3) as the within-participants factor was conducted for emotional valence scores.

For discernment of the number of flashes, error rates, and the d-prime (*d’*), and criterion (*c*) scores were calculated for each condition according to signal detection theory^42^. By using signal detection theory, discrimination performance can be separated into perceptual sensitivity (*d’*) and response bias (*c*). D-prime and criterion scores were calculated as follow: *d’* = *z*(H) – *z*(FA) and *c* = −0.5 × [*z*(FA) + *z*(H)], where *z*(p) denotes the inverse of the cumulative normal distribution corresponding to response rate *p*, and H and FA denote ‘hit’ (the correct response of two flashes when two flashes were presented, so hit could occur only in 2-flashes condition) and ‘false-alarm’ (an incorrect response of two flashes when one flash was presented, so false alarm could occur only in 1-flash condition). The *d’* and *c* were calculated separately when no-beep, 1-beep, or 2-beeps were presented under each face/shape condition same as previous studies^25,43^. If participants correctly discriminated between one and two flashes, then a high *d’* score would be obtained. Additionally, if participants’ responses were not biased for either one or two flashes, then the *c* score would be 0. On the other hand, negative *c* scores indicate a two-flash-directed bias; in contrast, positive *c* scores indicate a one-flash-directed bias. The fission illusion increases the false-alarm rate in 2-beeps conditions and fusion illusion decreases the hit rate in 1-beep conditions. Therefore, when the fission and fusion illusions occurred, the *d’* scores were expected to be low for the 2-beeps and 1-beep conditions compared to the no-beep condition^25,43^. Furthermore, *d’*-illusion and *c*-difference scores were calculated by subtracting *d’* and *c* scores with 1-beep/2-beeps from those with no-beep, respectively. The magnitudes of illusion and bias could be directly compared in fission and fusion illusions by using these scores^44,45^. A one-way ANOVA with face/shape (3) as the within-participant factor was separately conducted for *d’*-illusion and *c*-difference in 1-beep (i.e., fusion illusion) and 2-beeps (i.e., fission illusion) conditions. Being a multiple comparison test, Shaffer’s modified sequentially rejective Bonferroni procedure by Donoghue’s S2 algorithm^40,41^ was used throughout the experiments. Additionally, the correlation between the amounts of changing valence and *d’*-illusion (calculated by subtracting the angry/downward-pointing triangle and happy/circle from the neutral/upward-pointing triangle on valence and *d’*-illusion) was investigated in both fission and fusion illusions.

## Data Availability

The datasets generated and analyzed during the current study are available from the corresponding author on reasonable request.
